# Young, Online and in the Dark: Scaling Up HIV Testing among MSM in ASEAN

**DOI:** 10.1371/journal.pone.0126658

**Published:** 2015-05-14

**Authors:** Thomas E. Guadamuz, Doug H. Cheung, Chongyi Wei, Stuart Koe, Sin How Lim

**Affiliations:** 1 Department of Society and Health, Faculty of Social Sciences and Humanities, Mahidol University, Nakhon Pathom, Thailand; 2 Center for Health Policy Studies, Faculty of Social Sciences and Humanities, Mahidol University, Nakhon Pathom, Thailand; 3 Department of Epidemiology, Harvard School of Public Health, Boston, Massachusetts, United States of America; 4 Department of Epidemiology and Biostatistics & Global Health Sciences, University of California San Francisco, San Francisco, California, United States of America; 5 ICM Pharma Pte Ltd, Singapore; 6 Center of Excellence for Research in AIDS, University of Malaya, Kuala Lumpur, Malaysia; University of Toronto, CANADA

## Abstract

**Background:**

Poor HIV testing uptake by MSM may be attributable to unique challenges that are localized in Southeast Asia.

**Objective:**

To characterize MSM who never tested for HIV, to identify correlates of never testing, and to elucidate the perceived barriers to HIV testing.

**Methods:**

The present study used data from the Asian Internet MSM Sex Survey (AIMSS) and restricted the analysis to 4,310 MSM from the ten member countries of the Association of South East Asian Nations (ASEAN).

**Results:**

Among MSM participants from ASEAN in our sample, 1290 (29.9%) reported having never been tested for HIV, 471 (10.9%) tested for HIV more than 2 years ago, and 2186 (50.7%) reported their last test date was between 6 months and two years ago, with only 363 (8.4%) of these men having been tested in the past 6 months. In multivariable logistic regression, younger MSM (age 15–22 years old [AOR: 4.60, 95% CI: 3.04–6.96]), MSM with lower education (secondary school or lower [AOR: 1.37, 95% CI: 1.03–1.83]), MSM who identify as bisexual or heterosexual (compared to gay-identified) (AOR: 1.94, 95% CI: 1.60–2.35), and MSM who had never used a condom with male partners (AOR: 1.61, 95% CI: 1.32–1.97) had higher odds of never been HIV tested. Main reason for not being tested was a low risk perception of HIV exposure (n = 390, 30.2%).

**Conclusion:**

Current HIV prevention response must not leave MSM “in the dark,” but instead meet them where they are by utilizing the Internet creatively through social media and smart phones. As ASEAN Economic Community (AEC) is quickly becoming a reality, so must there be an equally fast and united response to slowing down the HIV epidemics among MSM in ASEAN.

## Introduction

HIV testing has become an integral part of combination prevention for men who have sex with men (MSM) because, in part, it is the entry point to the care continuum, and as such, it allows for timely linkage to HIV care and treatment, thus decreasing morbidity, mortality and onward HIV transmission [1,2]. In practice, however, HIV testing among MSM in Asia remains low, and actually lags behind other key populations. In Thailand, for example, testing prevalence has been reported to be 25% among MSM, compared with 50–60% among female and male sex workers [3]. HIV testing uptake is generally suboptimal among MSM in several other Southeast Asian countries, not only Thailand [4–11]. When compared to other lower and middle-income countries globally, Southeast Asian countries tend to have the lowest proportion of recently tested MSM, with a mere 20% of MSM having been tested for HIV in the past 12 months [12]. In addition, condom use reported by these men was also among the lowest, when compared with other regions [3]. These findings highlight the urgent need to increase HIV testing uptake among MSM in Southeast Asia.

Poor HIV testing uptake by MSM may be attributable to unique challenges that are localized in Southeast Asia. For example, despite free and low-cost HIV testing available in several countries in the region, there are still barriers to testing that are rooted in stigma and discrimination related to being HIV positive and being a sexual minority. These issues are manifested in the punitive laws against MSM and people living with HIV [13,14] as well as in the unfair treatment or discrimination by healthcare personnel [4,15]. Major barriers that prevent Asian MSM from testing include lack of knowledge about HIV testing costs and locations, lack of knowledge on the benefits of early treatment, perceiving oneself as having low HIV risk, and having misconceptions about HIV (e.g., HIV is a fatal illness/death sentence) [4–6,15].

In order to inform relevant public policies, we chose to analyze HIV testing at the regional level—ASEAN. The Association of Southeast Asian Nations (ASEAN) was established in 1967 to promote regional socio-political stability and economic growth and now includes 10 countries in Southeast Asia—Brunei, Cambodia, Indonesia, Laos, Malaysia, Myanmmar, the Philippines, Singapore, Thailand and Vietnam. Recently, leaders from ASEAN pledged to prevent and reduce new HIV infections and provide care, treatment, and support to key populations [16]. With the launch of the ASEAN Economic Community (AEC) to transform economies of the 10 countries into a single market in 2015, it is expected that this economic integration will require even more coordinated and concerted efforts in addressing public health problems such as HIV prevention, care and treatment. Currently, few studies have examined HIV testing and its correlates among MSM across ASEAN due, in part, to the difficulty of reaching MSM across these countries. Consequently, HIV testing behaviors among MSM have not been well understood.

Using data from a large online survey conducted among sexually-active MSM living in the member countries of ASEAN, the objectives of this study are to 1) characterize MSM who never tested for HIV, 2) identify correlates of never testing, and 3) elucidate the perceived barriers to HIV testing. These findings will inform HIV prevention programming and outreach to increase HIV testing among MSM in ASEAN.

## Materials and Methods

The present study used data from a large Internet survey of MSM in Southeast Asia. Details of the recruitment procedures and measures have been reported elsewhere [17]. Briefly, Fridae.com, a popular gay-oriented website in Asia, partnered with 40 AIDS community-based organizations (CBOs) and implemented an online survey—called the Asian MSM Sex Survey (AIMSS)—between January 1^st^ and February 28^th^, 2010. Banner advertisements were posted on Fridae.com. Emails were sent out to listserv members of collaborating CBOs. MSM who were 18 years or older were invited to complete the online survey. Participants anonymously provided online informed consent before proceeding to the questionnaire, available in English and 9 Asian languages and dialects (Bahasa Indonesa, Bahasa Melayu, Cantonese, Chinese Simplified, Chinese Traditional, Japanese, Tagalog, Thai, and Vietnamese). Participation in the study was anonymous, voluntary and no incentives were offered. During the two-month period, 13,883 men completed the online questionnaire with a response rate of 56.1%. Of those, we restricted our analytic sample to respondents who were currently living in ASEAN-member countries (N = 5661, 40.78%), we then further excluded 9 (0.16%) who were under the age of 18, 588 (10.4%) who never had anal sex, 855 (15.1%) who did not have a male sexual partner in the past 6 months, 40 (0.71%) who identified as female, 39 (0.69%) who identified as intersex, and 227 MSM (6.01%) with reported HIV seropositive status (these were not mutually exclusive categories). Our final analytic sample included 4,310 MSM.

### Measures

Participants were asked when they had their most recent voluntary HIV test (“never tested,” “in the last 6 months,” “6 to 12 months ago,” “1–2 years ago,” “more than 2 years ago”). If participants responded to “never tested,” they were further asked about the main reasons why they had not been tested. Demographic data such as age, country of residence, education, employment, marital status, and sexual orientation were collected. Participants self-reported HIV-related behaviors in the past six months, including number of male sex partners, frequency of condom use with all male partners, frequency of alcohol use and/or drug use prior to sex, and whether they had paid for sex or had sold sex to other men. Moreover, participants were asked about venues used to meet other men for sex, history of sexually transmitted infections (STI) or any STI symptoms in the past 6 months, perceived HIV risk (5-point Likert scale from ‘very low’ to ‘very high’) and number of gay friends (as a proxy to gay community connectedness [18,19].

### Statistical analysis

First, we aimed to characterize HIV testing among MSM from ASEAN-member countries. To accomplish this, we calculated the proportions of those who had never been tested by demographic variables (age, country of residence, education, employment, sexual orientation, etc), sexual risk behaviors in the past 6 months, and self-reported STI symptoms in the past 6 months. We then compared the odds of never HIV tested by these variables. Variables associated with odds of never tested for HIV (*p* ≤ 0.1) in the bivariate analysis or were theoretically relevant from the literature were included in the multivariable logistic regression model. We fitted candidate variables using a modified purposeful modeling approach [20]. We kept variables in the multivariable model based on the improvement of the AIC criterion, and removed variables with the highest insignificance level above a cut off *p* > 0.2 from the model. However, variables were kept if their removal changed the beta coefficients of other covariates by at least 15%. Multicollinearity was then checked using a variance-partitioned correlation matrix, variables with a correlation coefficient ≥ 0.70 were re-scaled or removed if theoretically redundant [21]. We also fitted interaction terms between sexual orientation, drug use and alcohol consumption in the past month, and number of gay friends. Signficant interactions were kept and insignficant main effects were removed. We then assessed the final model fit using the Hosmer and Lemeshow Goodness-of-Fit test.Sensitivity analysis was also conducted on the final model by multiple imputation (Markov chain Monte Carlo [MCMC] method, imputed copies = 5), assuming missing at random (MAR). To inform HIV testing outreach, we reported the frequencies and proportions on reasons for having never been tested for HIV among participants with an absence of any HIV testing history. All analyses were conducted in SAS version 9.3 with 0.05 critical values for hypothesis testing. Mahidol University and the University of California, San Francisco committees on human subjects research approved the analysis.

## Results

Among MSM participants from ASEAN-member countries in our sample, 1290 (29.9%) reported having never been tested for HIV, 471 (10.9%) tested for HIV more than 2 years ago, and 2186 (50.7%) reported their last test date between 6 months and two years ago, with only 363 (8.4%) of these men having been tested in the past 6 months. Similar to our prior publications with varying inclusion criteria, a majority of our participants were age 29 years or older, university educated, full-time employed, single and gay identified [17].

### Characteristics of MSM who had never been HIV tested

Table 1 shows the demographic and behavioral factors associated with HIV testing MSM who were a student at the time of survey assessment (47.8%), were unemployed (41.2%), were between age 18 and 22 years (63.0%), age 23 and 28 years (42.8%), and identified as bisexual (45.0%) or heterosexual/other (42.0%) had the highest frequency of having never been tested for HIV. Moreover, MSM from Southeast Asian countries reported varying proportions of having never been tested for HIV: the Philippines (55.8%), Indonesia (43.2%), Vietnam (33.8%), Malaysia (34.4%), Singapore (23.3%), Thailand (21.4%), and a pooled category of those residing in Brunei, Cambodia, Laos, and Myanmar (19.0%). In addition, we observed a clear descending gradient for those who reported one male sex partner (34.7%), two to five sex partners (31.3%) and six or more sex partners (22.9%) in the past 6 months (Crochran-Armitage trend test: z = 6.27, p<.001); and those who had never used condoms with male partners (37.7%), partial condom use (28.7%), and those who always used a condom (23.6%) in the past 6 months (Crochran-Armitage trend test: z = -3.28, p = .001); and those with no gay friends (46.9%), few (39.5), some (30.3%) and mostly (20.6%) gay friends (Crochran-Armitage trend test: Z = -4.00, p<0.001). Morevoer, those who have never been tested were more likely to report finding sexual partners using the Internet (32%, OR: 1.29, 95% CI: 1.06–1.58). Other factors associated with an absence of any HIV testing history in the bivariate analyses were country of residence, age, education, student status, marital status, sexual orientation, alcohol consumption prior to sex in the past 6 months, any drug use in the past 6 moths, and any self-reported STI diagnosis in the past 6 months.

**Table 1:** Factors associated with never tested for HIV among an online sample of 4,310 men who have sex with men (MSM) from ASEAN countries.

	Total	Never tested for HIV, n = 1290				
	N	n	(%)	OR	95% CI	
**Country**						
**The Philippines**	255	142	55.7	4.62	3.40	6.28
**Indonesia**	396	165	41.7	2.63	2.01	3.44
**Vietnam**	133	45	33.8	1.88	1.26	2.81
**Malaysia**	1199	412	34.4	1.93	1.55	2.39
**Singapore**	1572	366	23.3	1.12	0.90	1.39
**Thailand**	697	149	21.4	Ref		
**Brunei, Cambodia, Laos, and Myanmar**	58	11	19.0	0.86	0.44	1.70
**Age**						
**18–22**	183	116	63.4	6.59	4.99	8.69
**23–28**	1090	467	42.8	2.61	2.31	2.95
**29+**	3037	707	23.3	Ref		
**Education**						
**None-Secondary School**	452	170	37.6	1.95	1.53	2.49
**University/Tertiary degree**	2655	825	31.1	1.42	1.09	1.86
**Post-graduate/Professional school**	1203	295	24.5	Ref		
**Employment**						
**Student**	632	302	47.8	2.75	1.53	4.95
**Full-time**	3164	825	26.1	1.02	0.63	1.65
**Retired**	378	107	28.3	Ref		
**Unemployed**	136	56	41.2	1.81	0.92	3.53
**Sexual orientation**						
**Gay**	3522	937	26.6	Ref		
**Bisexual**	738	332	45.0	2.37	2.06	2.71
**Heterosexual or other** ^**a**^	50	21	42.0	—		
**Marital status**						
**Single**	3689	1137	30.8	1.64	1.45	1.86
**Married-Same sex**	396	91	23.0	Ref		
**Married-Opposite sex**	136	43	31.6	1.77	1.35	2.32
**Divorced or Widowed**	89	19	21.4	0.89	0.46	1.71
**Number of male sex partner in the past 6 months**						
**6+**	1186	272	22.9	Ref		
**2–5**	1907	596	31.3	1.78	1.49	2.13
**1**	1217	422	34.7	1.53	1.29	1.80
**Sex with any commercial sex workers, past 6 months**						
**Yes**	842	232	27.6	0.87	0.73	1.02
**No**	3468	1058	30.5	Ref		
**Received money to have sex with a man, past 6 months**						
**Yes**	346	94	27.2	0.86	0.68	1.11
**No**	3964	1196	30.2	Ref		
**Primary place to find sex partners, past 6 months**						
**Internet**	2666	853	32.0	1.29	1.06	1.58
**Bars, dance parties and friends**	731	191	26.1	Ref		
**Sex clubs, gay saunas and cruising spots**	915	247	27.0	0.94	0.82	1.07
**No. of gay friends**						
**Mostly**	1357	280	20.6	Ref		
**Some**	1783	541	30.3	1.68	1.42	1.98
**Few**	1074	424	39.5	2.51	2.10	3.00
**None**	96	45	46.9	3.39	2.23	5.18
**Condom use with all male partners, past 6 months** ^**b**^						
**Always**	1551	366	23.6	Ref		
**Sometimes**	1312	377	28.7	1.31	1.10	1.54
**Never**	769	290	37.7	1.96	1.63	2.36
**Consumed alcohol prior to sex, past 6 months**						
**Once or a few times per week**	1305	340	26.1	1.55	1.16	2.07
**Weekly to monthly**	372	69	18.56	Ref		
**Never**	2633	881	33.5	2.21	1.68	2.90
**Any drug use, past 6 months**						
**Once or a few times per week**	519	112	21.6	1.65	1.02	2.67
**Weekly to monthly**	168	24	14.3	Ref		
**Never**	3623	1154	31.85	2.80	1.81	4.35
**Any self-reported STIs, past 6 months**						
**Yes**	408	94	23.0	0.68	0.53	0.86
**No**	3902	1196	30.7	Ref		

^a^Collapsed with bisexual identity for subsequent analysis

^b^Sum not equal to total sample size due to missing responses

### Correlates of never been HIV tested

In our multivariable model (see Table 2), participants from the Philippines (AOR: 3.08, 95% CI: 2.15–4.43) and Indonesia (AOR: 1.89, 95% CI: 1.38–2.60), compared to Thailand, had higher odds of having never been HIV tested. However, men from Singapore (AOR: 0.68, 95% CI: 0.53–0.88), compared to Thailand, had lower odds of never been HIV tested after adjustment. Compared with older men, those who were between age 18 and 22 years (AOR: 5.55, 95% CI: 3.82–8.06) and those who were between age 23 and 28 years (AOR: 2.22, 95% CI: 1.87–2.65) had higher odds of having never been HIV tested. Bisexual, heterosexual or other identified men (AOR: 1.65, 95% CI: 1.35–2.01), compared to gay identified men, had higher odds of never been HIV tested. And compared to those with mostly gay friends, those with some (AOR: 1.40, 95% CI: 1.17–1.68), few (AOR:2.02, 95% CI: 1.66–2.48) and none (AOR: 2.57, 95% CI: 1.63–4.06) had higher odds of never been tested for HIV. In terms of past 6 months’ risk behaviors, men who had never used a condom with male partners (AOR: 1.58, 95% CI: 1.28–1.94), compared to those who always used a condom; those who never consumed alcohol prior to sex (AOR: 1.39, 95% CI: 1.01–1.92), compared to those who consumed more moderately; had higher odds of never been tested for HIV. Those who reported no recreational drug use (AOR: 2.17, 95% CI: 1.32, 3.58), compared to those with moderate use, had higher odds of having never been HIV tested. Men with any self-reported STI diagnosis (AOR: 0.75, 95% CI: 0.56–0.99), compared to those with none, had lower odds of never been tested for HIV. However, none of the interaction terms included were significant. Sensitivity analysis with imputed values did not show substantial influence on effect estimates, compared to the complete-case model (see S1 Table).

**Table 2:** Independent correlates of never tested for HIV among an online sample of men who have sex with men (MSM) from ASEAN countries (N = 3632)^a^.

Country	AOR	95% CI	
**The Philippines**	3.08	2.14	4.43
**Indonesia**	1.89	1.37	2.60
**Vietnam**	1.07	0.65	1.76
**Malaysia**	1.24	0.96	1.60
**Singapore**	0.68	0.53	0.88
**Thailand**	Ref		
**Brunei, Cambodia, Laos, and Myanmar**	0.59	0.28	1.24
**Age**			
**18–22**	5.55	3.82	8.06
**23–28**	2.22	1.87	2.65
**29+**	Ref		
**Sexual orientation**			
**Gay**	Ref		
**Heterosexual/ Bisexual**	1.65	1.35	2.01
**No. of gay friends**			
**Mostly**	Ref		
**Some**	1.40	1.17	1.68
**Few**	2.02	1.66	2.48
**None**	2.57	1.63	4.06
**Condom use with male partners, past 6 months**			
**Always**	Ref		
**Sometimes**	1.10	0.92	1.33
**Never**	1.58	1.28	1.94
**Consumed alcohol prior to sex, past 6 months**			
**Once or a few times per week**	1.10	0.79	1.53
**Weekly to monthly**	Ref		
**Never**	1.39	1.01	1.92
**Any drug use, past 6 months**			
**Once or a few times per week**	1.68	0.98	2.88
**Weekly to monthly**	Ref		
**Never**	2.17	1.32	3.58
**Any self-reported STIs, past 6 months**			
**Yes**	0.75	0.56	0.99
**No**	Ref		

^a^The model also adjusted for educational status and no. of male sexual partners in the past 6 moths.

Likelihood Ratio test: X^2^ = 510.1, df = 23, p<0.001

Max-rescaled (pseudo) R-Square = 0.188

Goodness-of-fit test: X^2^ = 5.65, df = 8, p = 0.687

### Barriers of HIV testing among MSM who have never been tested for HIV

Among participants who have never been tested for HIV, the main reason for not being tested were having a low risk perception of HIV exposure (n = 390, 30.2%) (see Table 3). More than 50% of these men included reasons for never been tested were related to HIV stigma, these included 19.3% who were afraid to find out that they were HIV positive and 12.4% who did not want to think about HIV in general or being HIV positive. Other reasons included concerns about HIV testing cost (3.0%), treatment (1.2%), and being afraid of needles (2.2%). In addition, more than 13% did not know where to get HIV tested.

**Table 3:** Reasons for never tested for HIV among an online sample of men who have sex with men (MSM) who never tested for HIV (N = 1290).

	n	%
**Low risk perception**		
**It's unlikely that I've been exposed to HIV**	390	30.2
**HIV stigma**		
**Fear of being HIV positive**	249	19.3
**I didn't want to think about HIV or being HIV positive**	159	12.3
**I don’t want to be seen by others in a HIV testing clinic**	53	4.1
**Afraid of stigma & discrimination**	46	3.6
**I am worried my name would be reported to the government if I was tested HIV positive**	44	3.4
**I'm afraid of losing job, insurance, housing, friends, family if people knew I was HIV positive**	40	3.1
**I don't trust the results to be confidential**	36	2.8
**It is a death sentence anyway and there is no hope**	20	1.6
**Financial concern**		
**HIV testing costs are too high**	39	3.0
**I cannot afford the treatment**	16	1.2
**Other**		
**I don't know where to get tested**	170	13.2
**I don't like needles**	28	2.2

## Discussion

Few studies have examined the prevalence and correlates of never testing for HIV, and even fewer studies have examined this among MSM. To our knowledge, this study provided, for the first time, HIV testing uptake information among MSM across ASEAN, particularly for countries where testing have not been previously reported (e.g., Indonesia, the Philippines, and Singapore). Our online sample of sexually active MSM suggests that lack of HIV testing may indeed be partly responsible for the high burden of HIV in Southeast Asia, with about one-third of the sample having never tested for HIV. This situation is made worst among MSM age 18–22 years where two-thirds have not been tested, further confirming previous offline findings from Thailand [22]. Targeted interventions that focus on young MSM age 18–22 are therefore needed across the region.

Moreover, our data show that having never tested was associated with never using condoms during anal sex and that one-third of those never tested did not feel they were at risk of HIV, suggesting that MSM who have never tested have riskier sex while not perceiving themselves or their partners as “risky” [23,24]. Men in our sample who were “heterosexual” or “bisexual” were also more likely to never test, suggesting that they may be afraid to access testing facilities in fear of stigma and discrimination [4,25,26]. For example, we found that about one fifth of the men in our sample who have never tested were afraid to find out if they were positive and, similar to other studies, more than one tenth of MSM did not know where to get tested. These findings confirm previous studies on the barriers of HIV testing [4,25].

Mathematical modeling has shown that if 80% of all persons with HIV were on treatment, global elimination of HIV will be possible in 50 years [1,27]. While this may be true in theory, in practice, it is almost impossible to achieve this goal when individuals engaging in high-risk behaviors are not getting tested [2]. In fact, evidence suggest that this strategy may even further increase ART cost if test-and-treat implementation is suboptimal and HIV prevalence is high [28]. Our finding has important implications, as Thailand just passed a law that will allow universal access to ARV, regardless of eligibility on CD4 count. This policy could require a rapid scale up of HIV testing in key populations, including MSM. However, recent data have shown that only 25% of Thai MSM have been tested in the past 12 months while 21.5% of Thai MSM from our online sample have never been tested for HIV [3]. Other Southeast Asian countries are likely to follow this legislation. And therefore, we need to reflect on the shortcomings of our current HIV prevention programs, and specifically on HIV testing uptake among MSM in Southeast Asia.

Previous findings have already established that MSM are online and that the online environment is where targeted HIV prevention and intervention activities, including HIV testing, should take place [10, 29–33]. This article extends previous findings by also suggesting that aside from being online, these men are also young (18–22 years) and “in the dark.” They are “in the dark” because (1) they engage in more unprotected sex, not as socially connected with other gay people, less likely to be “out,” and possibly more likely to be stigmatized for their sexual orientation; and (2) they live in countries with limited surveillance and research activities for MSM populations (e.g., the Philippines and Indonesia). To our knowledge, there are no online prevention or intervention activites to increase HIV testing that target MSM in ASEAN that have been properly evaluated and published in the global literature. However, there was recently a community-level online interventon conducted in Taiwan that used Internet peer opinion leaders (iPOL) that demonstrated a significant increase in HIV testing at 6 months (43.9% testing prevalence among MSM in the intervention arm versus 22.3% among MSM in the control arm, *p*<.001) and consistent condom use with online partners (34.2% consistent condom use prevalence among MSM in the intervention arm versus 26.2% among MSM in the control arm, *p* = .004) [10]. Further research are therefore needed to develop and evaluate tailored innovative online interventions appropriate for the needs of MSM in ASEAN, so that these men will no longer be “in the dark.”

There are limitations in this study. Since this is a cross-sectional survey, temporality is not established. Voluntary HIV testing and counseling uptake may also influence MSM’s subsequent behaviors. Further, our findings may not necessarily be generalizable to all MSM in ASEAN due to the lack of a probability-based sampling frame and because the requirement of Internet access may exclude some MSM from the sample. And while this survey reported barriers to testing, it did not include promoting factors, which have been reported in other studies among MSM [4,32]. Finally, there were small numbers of participants from several countries in ASEAN (Brunei (n = 27), Cambodia (n = 27), Mynamar (n = 10) and Laos (n = 8)). Despite these limitations, this is the first time HIV testing has been compared between member countries of ASEAN, such cross-country assessment is only possible through online recruitment.

## Conclusions

While this study did not specifically assessed MSM’s acceptability to obtain HIV prevention messages via social media and geo-spatial networking smartphone applications (apps), a recent study from Vietnam has demonstrated reasonable acceptability among online MSM [11]. Therefore, HIV prevention efforts may do well by utilizing the Internet creatively through social media and smart phones, and by collaborating with geo-spatial apps popular in the local setting [32,34]. Moreover, utilizing existing conceptualizations of HIV risks among MSM may be one way to contextualize HIV testing barriers and facilitators like the recently published studies grounded in the syndemics framework [19] from Thailand [35] and Vietnam [36]. As AEC is quickly becoming a reality, so must there be an equally fast and united response to slowing down the HIV epidemics among MSM in ASEAN. These findings can better inform regional and national policies of member countries in ASEAN.

## Supporting Information

S1 Table Independent correlates of never tested for HIV among an online recruited sample of men who have sex with men (MSM) from ASEAN countries based on imputed values (N = 4310). (DOCX)
