# Supplementary material for: Young, Online and in the Dark: Scaling Up HIV Testing among MSM in ASEAN
Source: PLoS One. 2015 May 14;10(5):e0126658. doi: 10.1371/journal.pone.0126658 (PMC4431680; doi:10.1371/journal.pone.0126658)
Supplement: S1 Table — (DOCX) [file pone.0126658.s001.docx]

| **Table S1. Independent correlates of never tested for HIV among an online recruited sample of men who have sex with men (MSM) from ASEAN countries based on imputed values (N= 4310)^a^** | | | |
| --- | --- | --- | --- |
| **Country** | AOR | 95% CI | |
| **The Philippines** | 4.08 | 2.93 | 5.66 |
| **Indonesia** | 1.93 | 1.44 | 2.58 |
| **Vietnam** | 1.33 | 0.86 | 2.05 |
| **Malaysia** | 1.35 | 1.07 | 1.71 |
| **Singapore** | 0.79 | 0.62 | 0.99 |
| **Thailand** | Ref |  |  |
| **Brunei, Cambodia, Laos, and Myanmar** | 0.61 | 0.30 | 1.25 |
| **Age** |  |  |  |
| **18-22** | 5.37 | 3.85 | 7.48 |
| **23-28** | 2.20 | 1.88 | 2.57 |
| **29+** | Ref |  |  |
| **Sexual orientation** |  |  |  |
| **Gay** | Ref |  |  |
| **Heterosexual/ Bisexual** | 1.57 | 1.31 | 1.87 |
| **No. of gay friends** |  |  |  |
| **Mostly** | Ref |  |  |
| **Some** | 1.42 | 1.18 | 1.70 |
| **Few** | 2.08 | 1.70 | 2.54 |
| **None** | 2.67 | 1.69 | 4.21 |
| **Condom use with male partners, past 6 months** | |  |  |
| **Always** | Ref |  |  |
| **Sometimes** | 1.10 | 0.90 | 1.34 |
| **Never** | 1.60 | 1.31 | 1.97 |
| **Consumed alcohol prior to sex, past 6 months** | |  |  |
| **Once or a few times per week** | 1.20 | 0.88 | 1.64 |
| **Weekly to monthly** | Ref |  |  |
| **Never** | 1.52 | 1.13 | 2.06 |
| **Any drug use, past 6 months** |  |  |  |
| **Once or a few times per week** | 1.61 | 0.97 | 2.67 |
| **Weekly to monthly** | Ref |  |  |
| **Never** | 2.05 | 1.28 | 3.26 |
| **Any self-reported STIs, past 6 months** |  |  |  |
| **Yes** | 0.69 | 0.53 | 0.90 |
| **No** | Ref |  |  |
|  | | | |
